# Public Opinions About Palliative and End-of-Life Care During the COVID-19 Pandemic: Twitter-Based Content Analysis

**DOI:** 10.2196/44774

**Published:** 2023-08-07

**Authors:** Yijun Wang, Emeka Chukwusa, Jonathan Koffman, Vasa Curcin

**Affiliations:** 1 Department of Population Health Sciences King's College London London United Kingdom; 2 Cicely Saunders Institute of Palliative Care King's College London London United Kingdom; 3 Wolfson Palliative Care Research Centre Hull York Medical School University of Hull Hull United Kingdom

**Keywords:** palliative care, end-of-life care, COVID-19, Twitter, public opinions

## Abstract

**Background:**

Palliative and end-of-life care (PEoLC) played a critical role in relieving distress and providing grief support in response to the heavy toll caused by the COVID-19 pandemic. However, little is known about public opinions concerning PEoLC during the pandemic. Given that social media have the potential to collect real-time public opinions, an analysis of this evidence is vital to guide future policy-making.

**Objective:**

This study aimed to use social media data to investigate real-time public opinions regarding PEoLC during the COVID-19 crisis and explore the impact of vaccination programs on public opinions about PEoLC.

**Methods:**

This Twitter-based study explored tweets across 3 English-speaking countries: the United States, the United Kingdom, and Canada. From October 2020 to March 2021, a total of 7951 PEoLC-related tweets with geographic tags were retrieved and identified from a large-scale COVID-19 Twitter data set through the Twitter application programming interface. Topic modeling realized through a pointwise mutual information–based co-occurrence network and Louvain modularity was used to examine latent topics across the 3 countries and across 2 time periods (pre- and postvaccination program periods).

**Results:**

Commonalities and regional differences among PEoLC topics in the United States, the United Kingdom, and Canada were identified specifically: cancer care and care facilities were of common interest to the public across the 3 countries during the pandemic; the public expressed positive attitudes toward the COVID-19 vaccine and highlighted the protection it affords to PEoLC professionals; and although Twitter users shared their personal experiences about PEoLC in the web-based community during the pandemic, this was more prominent in the United States and Canada. The implementation of the vaccination programs raised the profile of the vaccine discussion; however, this did not influence public opinions about PEoLC.

**Conclusions:**

Public opinions on Twitter reflected a need for enhanced PEoLC services during the COVID-19 pandemic. The insignificant impact of the vaccination program on public discussion on social media indicated that public concerns regarding PEoLC continued to persist even after the vaccination efforts. Insights gleaned from public opinions regarding PEoLC could provide some clues for policy makers on how to ensure high-quality PEoLC during public health emergencies. In this post–COVID-19 era, PEoLC professionals may wish to continue to examine social media and learn from web-based public discussion how to ease the long-lasting trauma caused by this crisis and prepare for public health emergencies in the future. Besides, our results showed social media’s potential in acting as an effective tool to reflect public opinions in the context of PEoLC.

## Introduction

### Background

Palliative and end-of-life care (PEoLC) aims to provide physical, psychological, and spiritual support to patients living with life-limiting conditions and terminal diseases to improve their quality of life and assist their families [1]. The COVID-19 pandemic led to >6 million deaths worldwide [2] and created an increased demand for PEoLC services [3], including providing relief to patients experiencing severe distress, supporting difficult decision-making, and delivering bereavement services to families and close friends. Recent evidence [4] suggests that approximately 15% of the patients with COVID-19 experienced moderate to severe pneumonia requiring inpatient care, and 1% to 2% needed intensive care unit admission and ventilator support [5]. The majority of these patients experienced severe symptoms, including dyspnea, agitation, cough, pain, and respiratory secretions, and many required palliative interventions [6]. It is estimated that nearly 16 million people experienced bereavement as a consequence of COVID-19, and 9.8% of them subsequently experienced prolonged grief disorder [7]. PEoLC also played a crucial role in providing bereavement support to this group of people [8,9]. However, PEoLC was under unprecedented strain and faced great challenges in delivering care during the COVID-19 pandemic [10,11]. A robust and up-to-date evidence base was needed to support policy-making to better allocate resources and meet the primary PEoLC needs of the public.

Public opinions refer to people’s views, attitudes, and emotions about public health events [12], and they play an influential role in the policy-making and policy adjustment processes within the realm of health care [13]. Empirical evidence has confirmed the utility of public opinions in guiding policy makers during the design of health care interventions because they provide insights into the public’s demands for health care [14,15]. In addition, public opinions serve as a valuable source of immediate feedback for policy makers, facilitating reflection on existing health care policies [16,17] and specific health interventions [18,19]. Therefore, public opinions may provide valuable insights concerning PEoLC and help policy makers to further improve PEoLC services during a pandemic [20,21]. To date, most studies have focused on professionals’ feedback about PEoLC services during the pandemic [22-25] and have not actively canvased the views of the public. Understanding the content of public opinions may additionally help researchers and policy makers when setting priorities and developing interventions.

More recently, a scoping review [26] identified the important role of social media in gauging public attitudes and emotions during the COVID-19 crisis; for instance, Abd-Alrazaq et al [27] examined 2.8 million tweets and identified 4 main topics on COVID-19: its origin, source, social and economic impact, and measures to curb its spread. Moreover, Zhao et al [28] demonstrated that social media data were comparable with those from traditional survey methods by analyzing Twitter posts from 2013 to 2019. Although some studies [29,30] used social media posts by PEoLC professionals to understand their experiences during the pandemic, few empirical investigations have been conducted into social media discussions on PEoLC by the general public.

### Objectives

This study used the Twitter microblogging platform to understand public opinions about PEoLC in the context of the COVID-19 pandemic. Specifically, the primary objective was to identify the top PEoLC topics discussed by the public in English-speaking countries during the pandemic. Our secondary objective was to understand in what ways the social media discussions about PEoLC changed after the start of the vaccination program and understand to what extent the stated benefits of the vaccine were reflected in public discourse, particularly the protective effect on PEoLC health professionals and patients [31-35].

## Methods

### Overview

**Figure 1:**
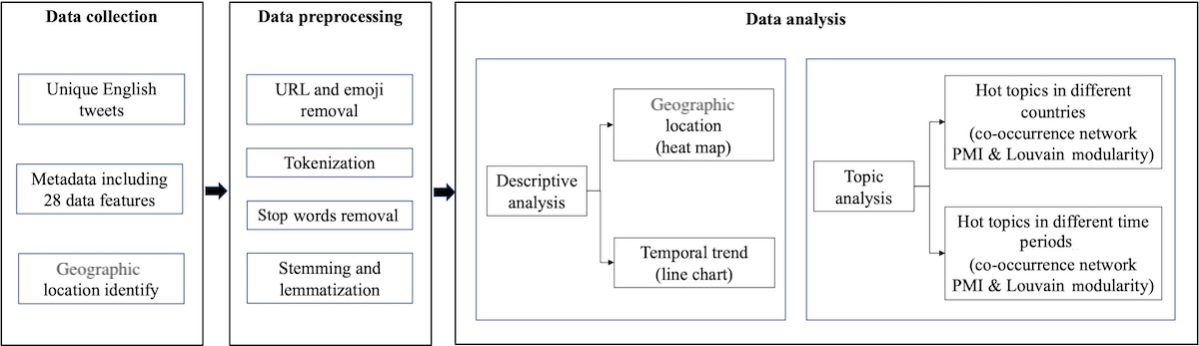
Flowchart of the methodology. PMI: pointwise mutual information.

This study adopted the methodology depicted in Figure 1 to understand frequently discussed topics about PEoLC during the pandemic and explore the changing patterns of the topics over time in 3 English-speaking countries: the United States, the United Kingdom, and Canada. These 3 countries were selected because they met the minimal size criteria of co-occurrence network analysis described in the following subsection of the *Methods* section on co-occurrence network, and tweets from them were representative of 80.96% (7951/9821) of all PEoLC English-language tweets with geographic location.

Relevant tweets, including their content and other metadata, were retrieved. Next, the retrieved tweets were cleaned before descriptive analysis to understand the spatial and temporal distribution characteristics of collected data. Topic modeling was then used to identify PEoLC-related topics in the 3 countries before and after the start of the vaccination programs. The start date of the vaccination program for the United States was December 10, 2020; for the United Kingdom, it was December 2, 2020; and for Canada, it was December 9, 2020.

### Data Collection Procedure

We used a large-scale COVID-19 Twitter chatter data set [36] comprising the tweet IDs of COVID-19–related tweets that was updated daily. The COVID-19 filtering keywords used by this data set were “COVID19,” “CoronavirusPandemic,” “COVID-19,” “2019nCoV,” “CoronaOutbreak,” “coronavirus,” and “WuhanVirus.” There have been >40 citations to this data set [36]. The full metadata of all tweets were retrieved through Twitter application programming interface v1.1 [37] using *Tweepy*, a Python library, filtering for English-language posts only.

As a second step, we designed a search filter to identify tweets that mentioned PEoLC among all COVID-19–related tweets and screen out irrelevant information (eg, related to COVID-19 but not concerned with PEoLC). For the sake of robustness, the search filter drew upon the experiences of previous empirical studies [38-40] and used the *snowball* search strategy [41] to identify new terms on Twitter. The filter design and initial results were validated through consultations with domain experts. The filter and key search terms for PEoLC-related tweets are presented in Textbox 1. The full metadata of identified PEoLC-related tweets were stored as JSON files.

**Key terms used for the palliative and end-of-life care (PEoLC) searching filter.**

“palliativetherapy,” “palliativeservice,” “palliativerehabilitation,” “palliativemedicine,” “palliativecareawareness,” “palliativecare,” “palliative therapy,” “palliative service,” “palliative rehabilitation,” “palliative medicine,” “palliative care,” “palliative assessment,” “palliative patient,” “palliative team,” “palliative staff,” “palliative_therapy,” “palliative_medicine,” “palliative_care,” “pallcare,” “palcare,” “pallmedicine,” “pallonc,” “pall care,” “oncology care,” “supportive_care,” “advanced care,” “terminal care,” “respite care,” “bereavement care,” “hospicecare,” “hospice care,” “hospice,” “eolcare,” “eolc,” “eol support,” “eol care,” “#eol,” “end of life,” “endoflifesupport,” “endoflifecare,” “end of life support,” “end of life care,” “end_of_life_care,” “ehospice,” “comfortcare,” “comfort care,” “cancercare,” “cancer care,” “supportivecare,” “supportive care,” “NCPGuidelines,” “NCP_Guidelines,” “NCPGuidelines,” “NCP Guidelines,” “hpmglobal,” “#hpm,” “#hapc,” “hospicecareweek,” “pallicovid,” and “covpall”

In total, 82,847,624 COVID-19–related tweets were retrieved during the period between October 1, 2020, and March 30, 2021, to gain a comprehensive understanding of public opinions regarding PEoLC before and after the commencement of the vaccination programs. Among these 82,847,624 COVID-19–related tweets, after filtering, 13,466 (0.02%) tweets related to PEoLC were identified.

For the spatial analysis of the retrieved tweets, user location was obtained through the user profile location where available. The post date was recorded for temporal analysis to compare posts across different time periods. In total, 9821 (72.93%) of the 13,466 PEoLC-related tweets included their geographic location.

### Data Preprocessing

A 4-step data preprocessing procedure was performed before data analysis. The first step comprised data cleaning by removing URLs and emojis from the original text using regular expressions. The second step involved tokenization or dividing the cleaned sentences into *terms* (tokens). The third step involved removing stop words (eg, “I,” “this,” “with,” and “or,” which do not add much meaning to a sentence). The fourth step represented stemming and lemmatization, generating the root forms of terms. After data preprocessing, the original text was transformed into separate terms for text mining.

### Co-Occurrence Network Based on the Pointwise Mutual Information Weighting Algorithm

A co-occurrence network based on the pointwise mutual information (PMI) weighting algorithm was used to visualize high-frequency terms in each topic to further explore the main content for each topic. The nodes and links in the co-occurrence network represented high-frequency terms and their associations. Popular topics in each country (the United States, the United Kingdom, and Canada) and each time period (before and after the implementation of the vaccination programs) were visualized through a co-occurrence network.

If 2 terms occurred in 1 tweet simultaneously, a link was made between them. The more frequently they appeared together, the greater the weight of the link and the thicker the line in the network. In the original network, the links were weighted based on the frequency in which the 2 terms occurred together. However, some pairs with greater weight appeared together much less frequently than they did individually. To avoid this situation, a PMI algorithm [42] was introduced to weight the link between terms (equation 1):




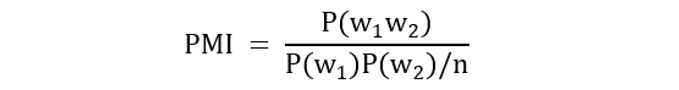


**(1)**

In equation 1, *P(w_1_w_2_)* represents the empirical probability of the terms *w_1_* or *w_2_* appearing in a tweet, *P(w_1_)* or *P(w_2_)* is the marginal probability of the terms *w_1_* or *w_2_* appearing in a tweet, and *n* is the total number of all tweets.

Compared with other topic models (eg, latent Dirichlet allocation [LDA] [43]), the co-occurrence network preserves more semantic information among terms. Especially with short-text data such as tweets, the performance of topic models such as LDA tends to be poorer than that with long-text data because of the sparse matrix [44]. Some topic models optimized for short-text data, such as the biterm topic model (BTM) and Bidirectional Encoder Representations from Transformers (BERT), have been tested on our data set and observed to exhibit poor differentiation among topics. A possible explanation of this might be the limited size of our data set, which ranged from 349 to 2394 tweets for each topic model, because there is a positive relationship between these model performances and data size [45]. Therefore, to extract key information based on preserving the original text information as much as possible, a co-occurrence network based on PMI was selected to conduct topic analysis. A suggested size for applying a co-occurrence network is >75 nodes [46].

### Modularity Based on the Louvain Method

Modularity based on the Louvain method [47] was used to explore latent topics in the co-occurrence network with a view to detecting community structure in the co-occurrence network of high-frequency terms. Terms in 1 community tend to have denser connections than terms in other communities. Therefore, these communities could be interpreted as popular topics if the terms in these communities also have semantic similarities. Newman [48] defined modularity as follows:




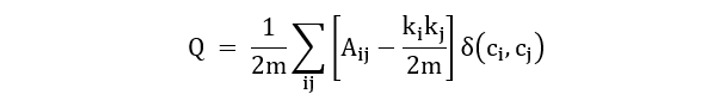


**(2)**

In equation 2, *A_ij_* is the link weight between term *i* and term *j*; *k_i_* and *k_j_* are the sums of the link weights attached to term *i* and term *j*, respectively; *m* is the sum of all link weights in this network; *c_i_* and *c_j_* are the communities of the terms; and 𝛿 is the Kronecker delta function [49].

The modularity optimization was achieved through the Louvain algorithm [47]. This optimization aimed to attain optimal community partitions with maximum modularity. The iteration automatically stops when there is no improvement in the value of modularity. Thus, the optimal division of communities, namely popular topics, was obtained, and the number of topics was settled.

### Ethical Considerations

This study represents a secondary analysis of an existing public Twitter data set [36]. As this data set is available in the public domain, an ethics review was not deemed necessary [50]. All identifiable information was removed from tweets to protect user privacy and anonymity. The data were collected and stored for no longer than was necessary for the purposes of this research paper.

## Results

### Geographic Location

**Figure 2:**
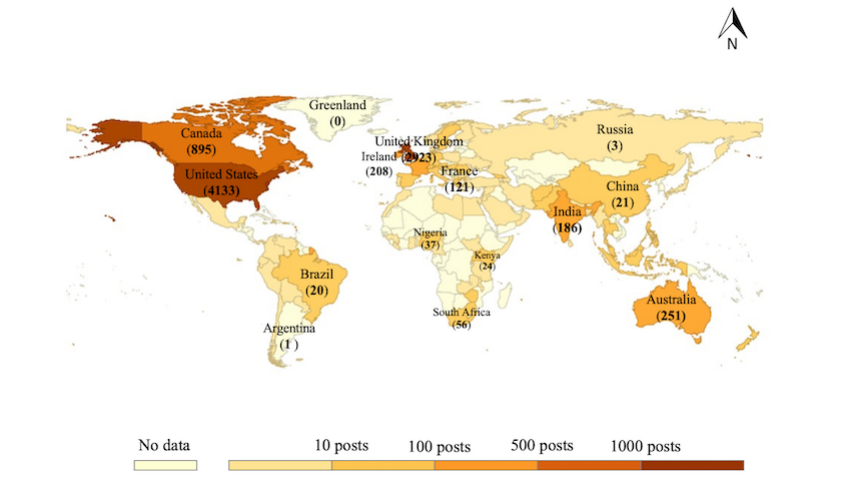
Global distribution of tweet numbers.

As only English-language tweets were collected, the geographic distribution was heavily skewed toward English-speaking countries in Figure 2.

**Figure 3:**
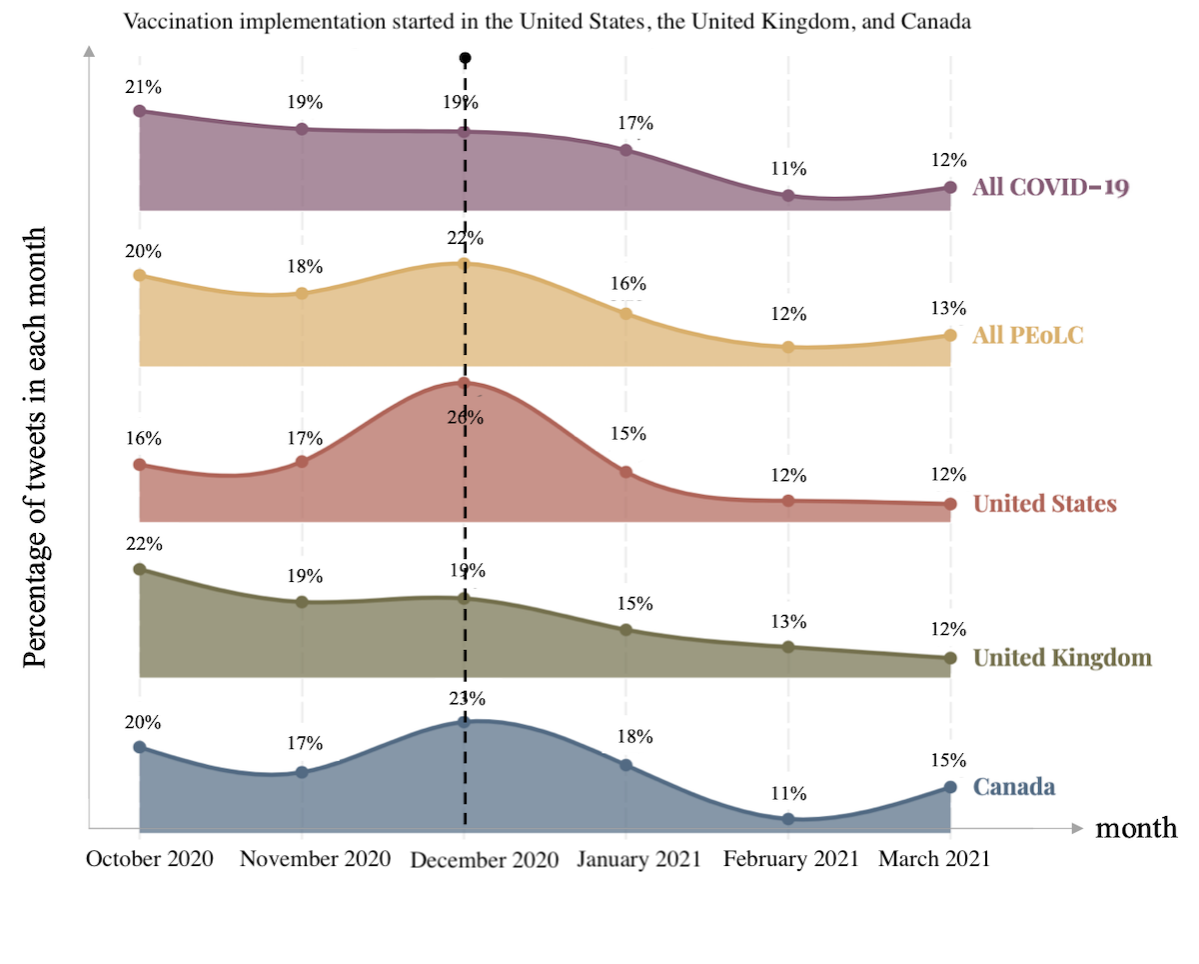
The monthly proportion of COVID-19 tweets and palliative and end-of-life care (PEoLC) tweets in the United States, the United Kingdom, and Canada.

Countries with a large number of PEoLC tweets were located in North America and Europe. The United States and the United Kingdom were the countries with >1000 tweets. There were 5 countries (Canada, Australia, Ireland, India, and France) with the number of tweets ranging between 100 and 1000, a total of 27 countries with the number of tweets ranging between 10 and 100, and 63 countries with the number of tweets ranging between 0 and 10. The detailed number of PEoLC tweets posted in each country was listed in Multimedia Appendix 1. The top 3 countries—the United States, the United Kingdom, and Canada—accounted for 80.96% (7951/9821) of all PEoLC tweets with geographic location. The monthly proportion of tweet numbers in these 3 countries is plotted in Figure 3.

Although the monthly percentage of COVID-19–related tweets showed a clear decrease, there was a surge in the number of PEoLC tweets in December 2020, which was also reflected in the number of PEoLC tweets posted in the top 3 countries. Furthermore, December 2020 was the month when these 3 countries officially commenced their vaccination programs.

### Co-Occurrence Networks in the United States, the United Kingdom, and Canada

To further understand the effect of the vaccination programs on PEoLC-related topics, we performed a before-and-after topic analysis on tweets from the United States, the United Kingdom, and Canada.

The United States granted emergency use authorization to the first COVID-19 vaccine on December 10, 2020 [51]. In total, 4133 PEoLC tweets were posted in the United States, of which 1739 (42.08%) were posted before the start of the vaccination program, and 2394 (57.92%) were posted after this date. Two co-occurrence networks were built based on tweets posted before and after the start of the vaccination program (Multimedia Appendix 2). Popular topics about PEoLC in the United States were summarized through network modularity (Table 1).

Popular PEoLC-related topics in the United States during the pandemic included personal experiences, research promotion, remote and in-home care, PEoLC for specified diseases, and vaccines for PEoLC stakeholders. There was no evident difference between topics before and after the implementation of the vaccination program except for the emergence of the topic of vaccines for PEoLC stakeholders.

The UK government authorized the first COVID-19 vaccine for use in the country on December 2, 2020 [52]. In total, 2923 PEoLC tweets were posted in the United Kingdom, of which 1226 (41.94%) were posted before the start of the vaccination program, and 1697 (58.06%) were posted after this date. Two co-occurrence networks were built based on tweets before and after the start of the vaccination program (Multimedia Appendix 3). Popular topics about PEoLC in the United Kingdom were summarized through network modularity (Table 2).

Popular PEoLC topics in the United Kingdom during the pandemic included fundraising for hospices, promoting research, thanks and praise, and care home. There was no evident difference between topics before and after the implementation of the vaccination program.

Health Canada authorized the first COVID-19 vaccine for use in the country on December 9, 2020 [53]. In total, 895 PEoLC tweets were posted in Canada, of which 349 (39%) were posted before the start of the vaccination program, and 546 (61%) were posted subsequently. Two co-occurrence networks were built based on tweets before and after the start of the vaccination program (Multimedia Appendix 4). Popular topics about PEoLC in Canada were summarized through network modularity (Table 3).

**Table 1:** Popular topics of palliative and end-of-life care (PEoLC) tweets posted in the United States before and after the start of the vaccination program.

Topics		Keywords	Examples (provided verbatim)
**Before**			
	Topic 1: personal experience	“mother,” “nursing home,” “test positive,” “ICU,” “friend,” “die,” “sick,” “old,” “end of life,” “family,” “sick,” and “pass”	My parents both died from COVID-19. My hero is my daddy’s hospice nurse. I was one of the lucky ones that were there day and night with my dad
	Topic 2: research promotion	“webinar,” “article,” “hpm,” “hapc,” “research,” “pallicovid,” “jamanetwork,” and “resources hub”	New resources added to the HPNA #COVID19 Resource Page this week from @Health_Affairs, @ANANursingWorld, @hospicebusiness, & @JAMANetwork. Check them out here
	Topic 3: remote and in-home care	“inhome,” “homehealth,” “telehealth,” “deliver,” “service,” and “require”	COVID-19 has exposed the vulnerability of nursing homes and shined a light on in-home care: a safer and cheaper option that delivers better outcomes
	Topic 4: PEoLC for specified disease	“oncology,” “kidney,” “breast cancer,” “patient,” and “dementia”	If you are looking for a giving option, check out the Coalition for Supportive Care of Kidney Patients
**After**			
	Topic 1: personal experience	“mother,” “nursing home,” “die,” “hospital,” and “family”	She moved her mother home with [a] hospice [nurse] from a nursing facility to help reduce the risk of COVID-19 exposure and maintain contact
	Topic 2: research promotion	“hapc,” “hpm,” “resources hub,” “team,” and “research”	Find resources and tools to help clinicians cope with moral distress, grief, and trauma in the CAPC COVID-19 Response Resources Hub
	Topic 3: vaccination	“vaccine,” “hospice staff,” “proud,” “woman,” “receive vaccine,” “frontline,” and “nursing home”	#Homehealth and hospice staff of all disciplines should be included in the first group of frontline care workers granted access to the #COVID19 vaccine

**Table 2:** Popular topics of palliative and end-of-life care tweets posted in the United Kingdom before and after the start of the vaccination program.

Topics		Keywords	Examples (provided verbatim)
**Before**			
	Topic 1: charity and fundraising	“charity,” “donation,” “fundraise,” “support,” “funding,” and “child”	Successfully made our #Covid19 #Contactfree Charity donation this evening in time for Christmas
	Topic 2: research promotion	“csilecture2020,” “hospiceuk,” “webinar,” “NHS,” “research,” “hospicecareweek,” “online,” and “bereavement”	The @cudippallmed team is planning a series of free webinars to support healthcare professionals in palliative & end of life care. Designed to inform, update & support you in issues arising during #Covid19
	Topic 3: thank and praise	“frontline,” “thank,” “work,” “support,” and “amazing”	Massive personal thank you to @TeessideHospice partners @RockliffeHall @CrathorneHall
	Topic 4: attention on care home	“visit,” “guidance,” “restriction,” “care home,” “family,” “government,” and “access”	Heartbreaking clip shows why care home visits must continue, especially at end of life # compassion #COVID19
**After**			
	Topic 1: charity to raise funding	“charity,” “donation,” “raise,” and “support”	Amazing @StChrisHospice is still caring for people living with life-limiting illnesses despite the coronavirus. Join in a Spooktacular #Quiz to raise vital funds
	Topic 2: research promotion	“hospicecare,” “peolc hospicecare,” “bereavement,” “webinar,” “report,” “research,” and “resource”	We are regularly updating our #Coronavirus webpage. It contains links to the latest official guidance and information, and a wealth of resources, including a guide for caring for someone dying at home. #peolc #hospicecare #Covid-19
	Topic 3: thank and praise	“thank,” “proud,” “great,” “frontline,” “NHS,” “huge,” “important,” “and hospiceuk”	Very proud of our wonderful #NorthEast hospices who have done amazing work through this period, and who are supported by many of our region’s clubs
	Topic 4: attention to care home	“die,” “involve,” “address location,” “residential,” “peak,” “hospital,” “receive,” and “vaccine”	Of the 2,768 total deaths involving COVID-19, 1,802 (65.1%) occurred in hospitals, 753 (27.2%) in care homes, 13 (0.5%) in hospices and 200 (7.2%) at residential addresses or other locations

**Table 3:** Popular topics of palliative and end-of-life care tweets posted in Canada before and after the start of the vaccination program.

Topics		Keywords	Examples (provided verbatim)
**Before**			
	Topic 1: Toronto palliative care	“Toronto palliative,” “Toronto,” “challenge,” “homeless,” “people,” “onpoli,” “need,” “Ontario,” and “challenge”	Toronto palliative patients who were homeless confronted new challenges due to COVID-19
	Topic 2: cancer-cannot-wait initiative	“cancercantwait,” “onpoli,” “care planning,” “ccsn,” “mpp,” “advocacy,” “virtual,” “discuss,” “disruption cancer,” “late,” and “delay”	Live Now! Advocacy Week Meeting! Discussion: How can we incorporate #cancercare in pandemic planning? With MPP @JR_Ottawa #onpoli #COVID19 #cancercantwait
	Topic 3: personal experience	“family,” “love,” “grief,” “caregiver,” “end of life,” “ltc,” “cancer patient,” “community,” and “experience”	My heart breaks for you & thousands more. I live in a country and was fortunate enough to spend 32 days in hospice with my beautiful sister before she died
**After**			
	Topic 1: new-normal-same-cancer care campaign	“newnormalsamecancer,” “thing normal,” “postpone,” “healthcare team,” “Canadian,” and “check”	The #NewNormalSameCancer campaign is a call to action for everyone living with cancer and pre-diagnosed cancer to reprioritize cancer care. Cancer won’t wait for #COVID19 to be over
	Topic 2: cancer-cannot-wait initiative	“2020 symposium,” “watch video,” “ccsn,” “disruption,” “webinar,” “cancerawareness,” “tune update,” and “open letter”	CADTH 2020 Symposium featured CCSN’s COVID-19 and the Disruption of Cancer Care in Canada. Watch the video! #cancercantwait #COVID19
	Topic 3: personal experience	“mother,” “visit,” “grief,” “family,” “hospital,” “end life,” and “die”	Feel so helpless for others! My mom-in-law died, not from COVID-19, but we couldn’t visit her
	Topic 4: vaccination	“vaccine,” “receive,” “staff,” “doctor,” “long,” “live,” “cancer patient,” and “family”	We are super excited to be part of the COVID-19 vaccine rollout! This is an important step in keeping not only our staff and hospice families safe, but our community as well

Popular PEoLC topics in Canada during the pandemic included the cancer-cannot-wait initiative, Toronto palliative care, the new-normal-same-cancer care campaign, personal experiences, and vaccines for PEoLC stakeholders. The new-normal-same-cancer care campaign appeared after the implementation of the vaccination program, an indication of the public’s ongoing concerns about cancer care. The vaccine was another topic that emerged after the implementation of the vaccination program in Canada.

Taken together, during the COVID-19 pandemic, PEoLC topics in the United States, the United Kingdom, and Canada showed some similarities as well as differences. Topics concerning cancer care (eg, the topics of research promotion in the United States and the United Kingdom and the cancer-cannot-wait initiative in Canada) and care facilities (eg, the topics of personal experience in the United States and Canada and attention on care homes in the United Kingdom) were commonly discussed in these 3 countries. The topic of charity and fundraising for PEoLC was specific to the United Kingdom. Except for the emerging topic of vaccination, there were no notable differences among PEoLC topics discussed before and after the implementation of the vaccination program in the United States, the United Kingdom, and Canada.

## Discussion

### Principal Findings

This study conducted an analysis of Twitter posts with the primary aim of understanding public opinions about PEoLC and the secondary aim of examining the impact of the vaccination program on public opinions regarding PEoLC. We observed that the public discussion on Twitter provided useful insights into the reflections of public concerns regarding PEoLC during the COVID-19 pandemic. Our results identified that cancer care and care facilities (eg, nursing homes, hospice homes, and care homes) were the 2 most frequently discussed topics in the United States, the United Kingdom, and Canada. Twitter users also shared their personal PEoLC experiences on the web, and this was more common in the United States and Canada. In addition, the vaccine became an increasingly popular topic to tweet about after the implementation of the vaccination program, with associated positive attitudes in the context of PEoLC. These findings broadly supported those of previous studies [54-59] and partially verified our hypothesis about using social media for performing public opinion studies in the context of PEoLC.

It may not be surprising that cancer care was one of the most frequently discussed PEoLC topics during the pandemic. Cancer continues to be one of the leading diagnoses for which palliative care is needed [60]. According to the Lancet Commission’s recent report on palliative care [61], cancer is responsible for the largest number of people experiencing serious health issues and is emblematic of palliative care needs. Cancer care may have emerged as one of the most popular PEoLC topics owing to the uncertainty in accessing and providing care brought about by the pandemic [62-64]. A large proportion of medical resources were devoted to the prevention, control, and treatment of patients with COVID-19, leaving patients with cancer in a more vulnerable situation [63]. Although a number of strategies [62,63] were proposed to ensure service quality and cope with the shortage in cancer care resources, higher exposure risk, and other potential issues, the situation faced by patients with cancer and caregivers was, nevertheless, adversely affected by the pandemic. On the basis of national survey results from the Canadian Cancer Survivor Network that aimed to understand the COVID-19 impact on 1502 patients with cancer between June 10, 2021, and July 4, 2021, delays in appointments and treatments affected >50% of the participants [54]. A US study in March 2020 [63] also implied that there was public dissatisfaction with the existing cancer care system during the COVID-19 pandemic because of unmet medical needs. Our study yielded results similar to those of the national surveys, supporting the concern that cancer care may have been negatively affected by the pandemic in the United States, the United Kingdom, and Canada. Specifically, the results identified that tweets related to cancer care typically comprised negative terms (eg, “delay,” “cancel,” and “disruption”) across all 3 countries, either before or after the implementation of the vaccination program. This suggests that the public discussion regarding cancer care did not change even after the implementation of the vaccination program. Alongside difficulties in accessing cancer care, the psychological stress caused by unpredictable and rapidly changing policies during the pandemic may represent another persistent problem for patients with cancer and caregivers [65]. It is possible that social media provided them with a platform and agency to share personal traumatic experiences. Specifically, “cancer cannot wait” was the message that was reiterated through all these related tweets. With the global health system switching back to normal operation, cancer care should rank highly on policy makers’ and health professionals’ agendas.

Twitter users shared their personal experiences about care facilities and home care when referring to PEoLC on social media platforms during the pandemic. Overall, 14.17% (1127/7951) of tweets were PEoLC-related tweets, mentioned synonyms for familial relationships (eg, “father,” “mother,” and “grandparent”) or friends and acquaintances (eg, “buddy,” “pal,” and “colleague”), which were categorized as *personal experience sharing* in this study. In these tweets, part of the negativity stemmed from the visiting restriction policy imposed on care facilities. National survey results in Ireland [55] and the Netherlands [56] also showed significantly lower well-being and increased sadness and fear among the relatives of care facility residents regarding the visiting restrictions during the pandemic. Although physical distancing was shown as being effective in curbing the spread of COVID-19, it has been previously established [66-68] that isolation may cause additional negative effects on the health status of care facility residents and exacerbate the mental stress of their family caregivers. Some family caregivers chose to move their dependents to their home instead of care facilities as a consequence of the very restrictive visiting policies, the exposure risk of the virus, and the work-from-home option [69,70]. However, owing to the shortage of medical resources and distance restrictions, home care for patients needing palliative care suffered from a lack of in-person nurse assistance. When delivering offline PEoLC is challenging, making full use of web-based resources may be a feasible solution (eg, holding workshops or providing information about home care skills for these caregivers). Some web-based information hubs [71,72] were developed to provide the public with home care resources and web-based consultation. Academic reports highlighted that some home caregivers reported inadequate health care knowledge and increased psychological stress during the pandemic [73]. Therefore, more efforts for enhancing in-home palliative care are still required.

Personal PEoLC experiences were shared on the web to share grief and seek spiritual or physical support during the pandemic. The emergence of web-based networks has profoundly changed how people experience dying and how they express their grief [74]. This was further transformed by the pandemic crisis and the related policies that restricted real-world communications. According to Statista global survey results [57], 44% of people spent more time on social media owing to the COVID-19 outbreak. The absence of traditional grief rituals that include saying goodbye as well as viewing and burying the body and the lack of physical social support during the pandemic led to people finding alternative means to express grief and seek support. We identified that social media provided individuals with the agency to share their expressions of grief during the pandemic, which, in the absence of more traditional sources of support, will have been beneficial and therapeutic [75,76]. The use of social media to share evocative messages was brought into sharper relief during the pandemic, and it could be argued that this was a means of normalizing the grief process and reducing the stigma surrounding death.

We also identified important geographic variations. Specifically, social media users in the United States and Canada were more likely than those in the United Kingdom to share personal experiences in the web-based community. The 2020 Commonwealth Fund International Health Policy Survey [58] reported that a greater proportion of people in the United States and Canada experienced stress, anxiety, or sadness that was difficult to cope with alone since the COVID-19 outbreak started compared with people in the United Kingdom. Social media may function as a communication channel for people to share personal feelings to relieve mental distress.

Within the PEoLC discussions on social media, the commencement of the vaccination program was associated with an increased volume of, at times, emotive discussions about vaccines, with the general public displaying generally positive attitudes. Although COVID-19 tweets decreased in number, PEoLC tweets reached their peak in December 2020, around the authorization of the COVID-19 vaccination programs, indicating that the public discussion about PEoLC responded to this policy. Other studies [31,77] have emphasized the importance of vaccination programs to PEoLC strategies during the pandemic, and our study supported this finding. In addition, our results indicated that the term “vaccine” was closely related to positive terms that included “thank,” “love,” “support,” “frontline,” and “proud” across all 3 countries after the implementation of the vaccination programs. This contradicted previous studies that identified vaccine hesitancy as being a common phenomenon in the United States, the United Kingdom, and Canada [17,78-80]. This study observed generally positive attitudes toward vaccines among those individuals who tweeted about PEoLC. Although some Twitter users expressed gratitude for receiving the vaccine, other users issued appeals for increasing the supply of vaccines to protect more health professionals working in PEoLC owing to the greater vulnerability of this group. The results of a Centers for Disease Control and Prevention [59] survey stated that COVID-19 vaccine coverage in care homes was much higher among residents than among care home staff. Future research needs to understand the potential reasons behind this phenomenon and whether the low vaccine coverage among staff is caused by vaccine hesitancy or a shortage in vaccine supplies.

### Implications for PEoLC

During the COVID-19 pandemic, because of the ensuing lockdowns and physical distancing policies, there was an increased use of social media to share public perceptions of public health events or policies regarding COVID-19 [81]. Among all kinds of topics, PEoLC was one of the foci of public attention, perhaps because of the increased number of deaths [82]. Many caregivers shared their worries or grief on social media platforms such as Twitter, which also proved to be a valuable resource for PEoLC providers to offer personalized services. Social media posts by caregivers and patients may be valuable for enhancing PEoLC services for the following reasons. First, as noted in a previous study [83,84], the content posted by caregivers or patients on social media platforms may assist PEoLC staff to understand their preferences and values when facing end-of-life issues and being unable to communicate in person. Second, our study suggests that social media posts may become an additional channel to help PEoLC professionals detect people in potential need because of the grief or other needs that they choose to share. Natural language processing methods have been used to develop targeted advertising of health services on social networks [85]. This may have applicability in the context of PEoLC. Depending on their preferences, PEoLC professionals may therefore wish to consider the potential of communicating with, and supporting, patients and their caregivers through social media.

Social media is currently underused in PEoLC research [86]. Beyond the potential for assisting professionals in improving personalized PEoLC, shared social media data may also act as a complementary research resource. A systematic review [87] identified 6 areas where Twitter may enhance health research, namely content analysis, surveillance, engagement, recruitment, as part of an intervention, and network analysis of Twitter users. Specifically, public bereavement experiences were explored during the pandemic, and volunteers were recruited offline to share their experiences [88-90]. Social media may therefore be used to extend recruitment beyond existing boundaries to achieve reach and enhance the diversity of participants currently recruited to both quantitative and qualitative studies in PEoLC.

One of the issues that emerged from our findings is the public concern about cancer care and in-home care during the pandemic. Advanced telehealth may be an efficient way to improve these kinds of PEoLC services in the face of an infectious disease outbreak [91]. Cancer care is an area that may benefit from integrating telehealth into home care services. The high infection risk of in-person clinic visits, travel restrictions, and personnel shortage were some of the major factors behind patient dissatisfaction during the COVID-19 pandemic that were explored through the cancer care discussion in a study [63]. Advanced telehealth is a cost-effective way to reduce infection risk and optimize service delivery through involvement in screening of patients with cancer, diagnosing monitoring, and providing telechemotherapy [91,92]. In addition, other PEoLC stakeholders may benefit from advanced telehealth for home care. Telehealth provides a solution to meet the growing demands of palliative care services across geographic regions with limited resources and outside of normal working hours through remote monitoring and support for patients who need palliative care and caregivers [93], which was in urgent need during the pandemic.

Although the COVID-19 pandemic has gradually become a new normal, and the pressure it brought to bear on the global health system has reduced, some studies [94-97] has indicated the persistent impact of the pandemic on global health. In this post–COVID-19 era, PEoLC professionals may wish to learn from public feedback and ease the long-lasting trauma caused by the crisis.

### Strengths and Limitations

This study has a number of strengths and limitations that affect the inferences that can be drawn from the results presented. A strength of this study is the use of social media, which can dramatically extend the reach of studies across multiple continents and countries. Another important strength is our innovative adoption of a semantics-based approach to examine the use of social media (compared with previous statistical-based topic analysis methods designed for large data volumes, eg, LDA, BTM, and BERT) to provide a useful exemplar for future works with relatively small data volumes. We observed that this semantics-based approach is more suitable when the volume of data does not reach tens of thousands. After comparison, our results showed that topic modeling based on a co-occurrence network and Louvain modularity demonstrated better performance on our data set.

Our study also has several limitations. First, our design was observational and exploratory owing to the limited representativeness of social media data. Social media users tend to be younger and more educated than nonusers [98], which may affect the generalizability of our findings. Consequently, we cannot draw firm conclusions but only interpret with caution our findings based on tweets. Studies that adopt other complementary research approaches (eg, surveys and interviews) are required to validate the findings from this study, including triangulating public opinions on Twitter. Second, we only included PEoLC tweets with COVID-19–related keywords to identify special public concerns about PEoLC during the pandemic. Without pre–COVID-19 data as a control group, it is difficult to identify issues that were exclusively associated with the outbreak. We applied a variety of methods to ensure that PEoLC-related keywords were included. Notwithstanding this, such a filter design may still omit some tweets that could reflect specified PEoLC needs of people with nonmalignant diseases caused by COVID-19. Future work should compare public concerns before and after the COVID-19 pandemic. To develop a definitive picture of PEoLC public opinions, additional studies will be needed to investigate PEoLC public opinions in non–English-speaking countries; the data collection and analytical methods proposed by this study should be drawn upon.

### Conclusions

This study aimed to collect and understand public opinions about PEoLC during the COVID-19 pandemic. We found that the public frequently discussed cancer care and care facilities when talking about PEoLC on Twitter. They also shared personal experiences about these PEoLC topics during the pandemic to express grief or seek support. The commencement of vaccination programs did not significantly affect the public discussion. However, positive attitudes toward the topic of vaccines for PEoLC stakeholders were detected. These findings are supported by those of other studies using traditional data and demonstrate the potential of using social media to understand public opinions relevant to PEoLC. Health professionals and policy makers may be able to use these findings to better understand public concerns and refine care services after the pandemic. More efforts should be made in the future to explore how public opinions on social media could better help PEoLC professionals to improve the end-of-life care experience of the public in a health emergency (eg, future pandemics).

## Appendix

Multimedia Appendix 1 Number of palliative and end-of-life care tweets posted per country.

Multimedia Appendix 2 Co-occurrence networks of palliative and end-of-life care tweets posted in the United States before and after the start of the vaccination program.

Multimedia Appendix 3 Co-occurrence networks of palliative and end-of-life care tweets posted in the United Kingdom before and after the start of the vaccination program.

Multimedia Appendix 4 Co-occurrence networks of palliative and end-of-life care tweets posted in Canada before and after the start of the vaccination program.

## Abbreviations

BERT: Bidirectional Encoder Representations from Transformers
BTM: biterm topic model
LDA: latent Dirichlet allocation
PEoLC: palliative and end-of-life care
PMI: pointwise mutual information

## Data Availability

All data generated or analyzed during this study are included in a published article [36] that is openly available at Zenodo [99].
