# Supplementary figures and images for: Public Opinions About Palliative and End-of-Life Care During the COVID-19 Pandemic: Twitter-Based Content Analysis
Source: JMIR Form Res. 2023 Aug 7;7:e44774. doi: 10.2196/44774 (PMC10408639; doi:10.2196/44774)

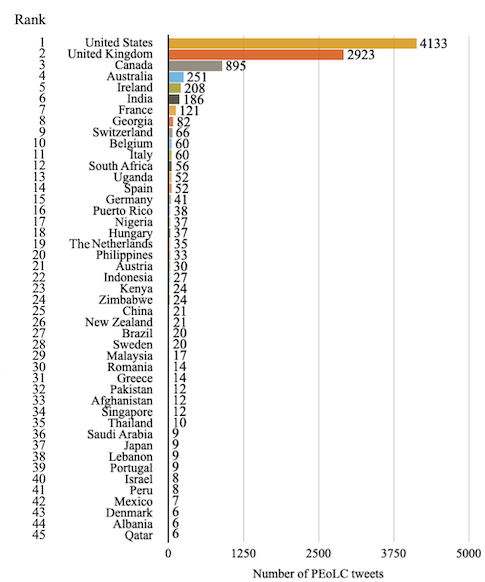

Supplement: Multimedia Appendix 1 [file formative_v7i1e44774_app1.png]

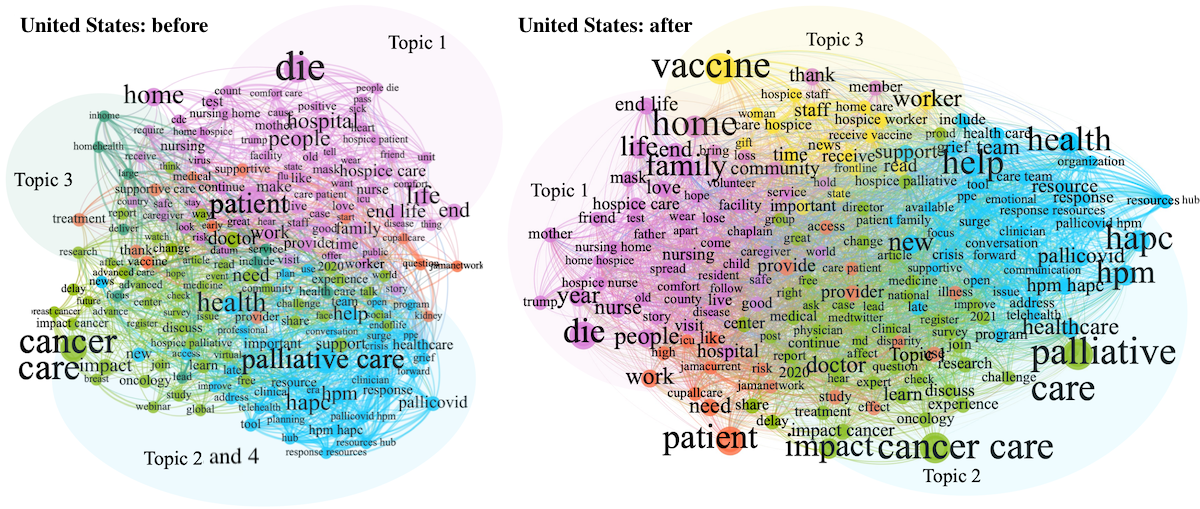

Supplement: Multimedia Appendix 2 [file formative_v7i1e44774_app2.png]

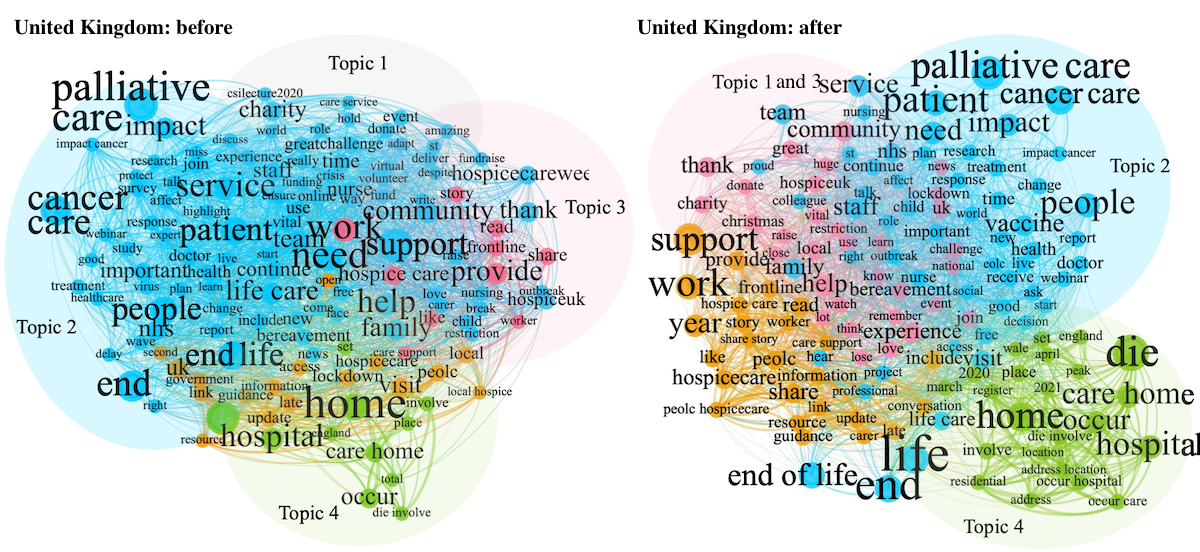

Supplement: Multimedia Appendix 3 [file formative_v7i1e44774_app3.png]

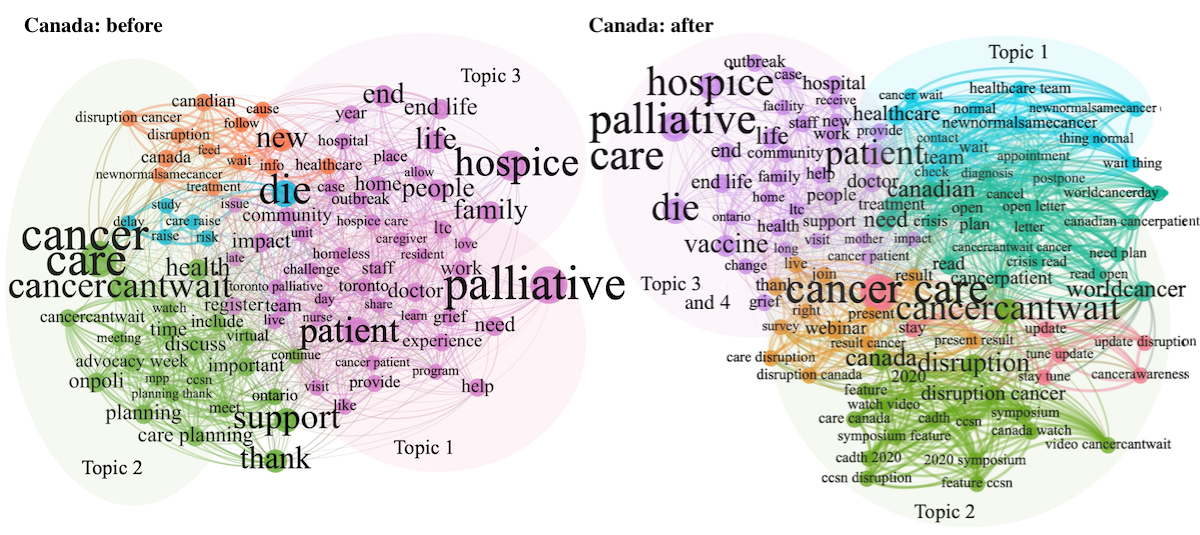

Supplement: Multimedia Appendix 4 [file formative_v7i1e44774_app4.png]
